# Patient Portal Functionalities and Uptake: Systematic Review Protocol

**DOI:** 10.2196/14975

**Published:** 2020-07-31

**Authors:** Abrar Alturkistani, Geva Greenfield, Felix Greaves, Shirin Aliabadi, Rosemary H Jenkins, Ceire Costelloe

**Affiliations:** 1 Global Digital Health Unit Department of Primary Care and Public Health Imperial College London London United Kingdom; 2 Public Health Policy Evaluation Unit Department of Primary Care and Public Health Imperial College London London United Kingdom; 3 Department of Primary Care and Public Health Imperial College London London United Kingdom

**Keywords:** personal health record, patient portal, electronic health records, online access, patient records, systematic review

## Abstract

**Background:**

Patient portals are digital health tools adopted by health care organizations. The portals are generally connected to the electronic health record of the health care organization and offer patients functionalities such as access to the medical record, ability to order repeat prescriptions, make appointments, or message the health care provider. Patient portals may be beneficial for both patients and the health care system. Patient portals can widely differ from one context to another due to the differences in the portal functionalities and capabilities and it is anticipated that outcomes associated with the functionalities also differ. Current systematic reviews report outcomes associated with patient portal uptake but do not explicitly specify the patient portal functionalities.

**Objective:**

The aim of this systematic review is to synthesize the evidence on health and health care quality outcomes associated with patient portal use among adult (18 years or older) patients. The review research questions are as follows: What kind of health outcomes do tethered patient portals and patient portal functionalities contribute to in adult patients (18 years or older)? and What kind of health care quality outcomes, including health care utilization outcomes, do tethered patient portals and patient portal functionalities contribute to in adult patients (18 years or older)?

**Methods:**

The systematic review will be conducted by searching the MEDLINE, EMBASE, and Scopus databases for relevant literature. The review inclusion criteria will be studies about adult patients (18 years or older), studies only about tethered patient portals, and studies with or without a comparator. We will report patient portal–associated health and health care quality outcomes based on the patient portal functionalities. All quantitative primary study types will be included. Risk of bias of included studies will be assessed using the Cochrane Collaboration’s tool for assessing risk of bias in randomized trials and the National Heart, Lung, and Blood Institute’s quality assessment tools. Data will be synthesized using narrative synthesis and will be reported according to the patient portal functionalities, country, disease, and health care system model.

**Results:**

Searches will be conducted in September 2019, and the review is anticipated to be completed by the end of June 2020.

**Conclusions:**

This systematic review will provide an overview of health and health care quality outcomes associated with patient portal use among adult patients, providing detailed information about the functionalities of the portals and their associations with the outcomes. The review could potentially help patient portal evaluation studies by providing insights into outcomes associated with the different functionalities of patient portals.

**Trial Registration:**

International Prospective Register of Systematic Reviews (PROSPERO) CRD42019141131; https://www.crd.york.ac.uk/prospero/display_record.php?RecordID=141131

**International Registered Report Identifier (IRRID):**

PRR1-10.2196/14975

## Introduction

Technology is affecting all aspects of health care systems. With the introduction of electronic health records (EHRs), patients are now able to access their medical records through patient portals, or personal health records (PHRs). Tethered patient portals or PHRs are connected to the EHR operated by the health care provider or organization and typically contain information about patient’s health records such as allergies, immunizations, medication, and upcoming appointments [1]. Some patient portals can also include information such as genetic data, preventative or customized medical advice [1], or offer functionalities such as ordering repeat prescriptions, messaging health care providers, and sharing health care record [2].

Patient portals are generally offered through the primary care providers, but can also be offered in hospital care, or during acute care [3]. The technology provides some benefits to patients and the health care system. For the health care system, portals may contribute to reducing phone call and visits [4], reducing emergency department visits [5], reducing hospital readmissions, improving the quality and efficiency of the health care system [6,7], and reducing health care service utilization in the long run and improving adherence to medical appointments [8]. For the patient, the use of patient portal may contribute to assisting in medical decision making [9], improving health care outcomes [6,10], improving adherence [11], improving patient- or person-centered care [4], improving patient satisfaction and increasing patient safety [12], and improving disease management and prevention [6,7]. In addition, patient portal functionalities such as viewing medical record may improve the relationship between patients and their providers [13]. Patient portals also make it possible to connect different emerging technologies such as wearable devices and mobile health (mHealth) technologies and collect the information in the patient record [14].

**Figure 1:**
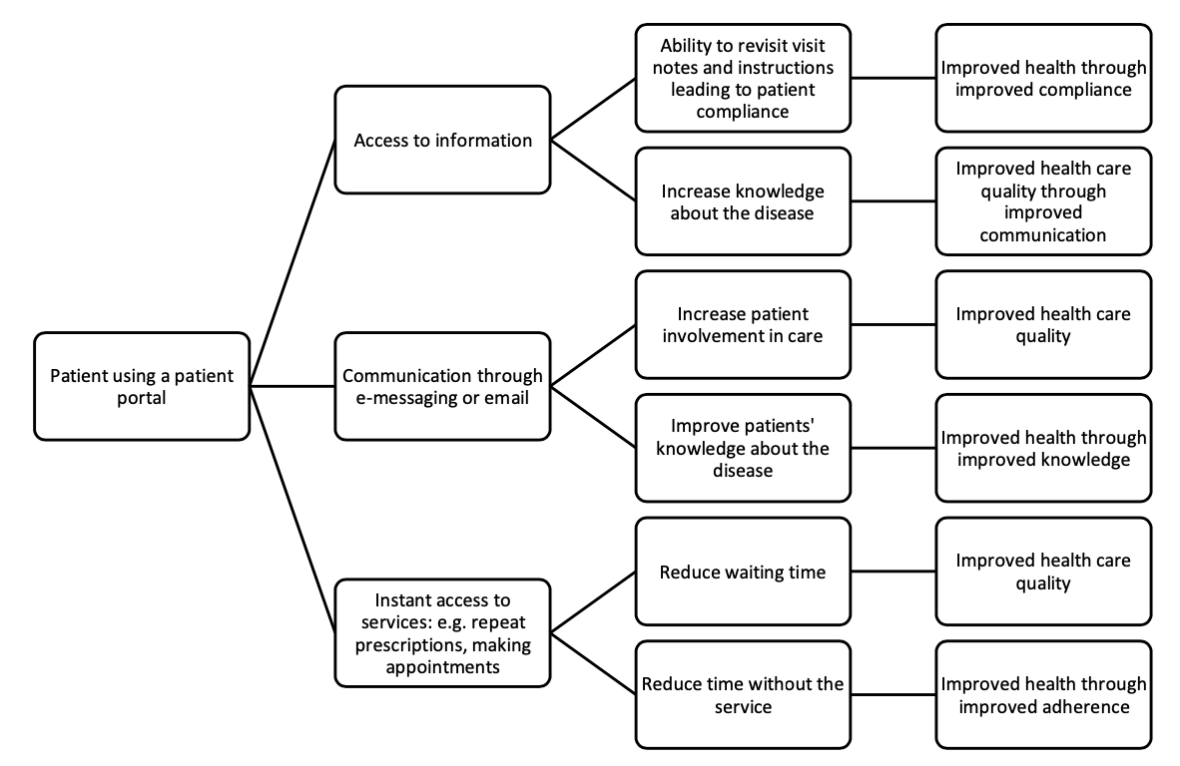
Theoretical framework of how patient portal use could lead to improved health and health care quality.

Although patient portals are associated with benefits to patients’ health and to the health care system, there are no definitive explanations about how they contribute to improved outcomes. A theoretical framework depicting how a patient portal access could improve health and health care quality experience is illustrated in Figure 1 using findings of qualitative studies. The benefits are often explained through the use of specific functionalities of the portal. For example, patients’ access to the health record with visit notes gives them ability to revisit their notes, which in turn improves their compliance with the care plan set out by the doctor [15,16] and also increases their confidence [17,18]. At the same time, access to the health record or test results gives the patients the chance to instantly and continuously review their record, increasing their knowledge about their condition [15] and improving their communication with the health care provider through increased ability to discuss their health care condition [15,16,18]. Other functionalities of patient portals such as e-messaging or secure messaging and patient education can improve patients’ involvement in their care [15] and their knowledge about their disease [19], respectively. Patients’ ability to refill medication through the portal allows them to efficiently refill medication, reducing the time they spend without medication, and as a consequence may improve their adherence and compliance with medication regime [17,20].

When reviewing patient portal studies it is important to consider possible sources of bias. Patient portal studies are often subject to bias due to the complex mechanisms involved between technology use and outcomes. Uptake usually varies between patients and often only registration on the portal is accounted for in studies. For example, while a meta-analysis reported a mean adoption rate of 51% (95% CI 42%-62%) from 40 studies about patient portals [21], a patient portal for laboratory results viewing with additional functionality of e-messaging received only 8.91% views out of the 208,635 tests released through the portal [22]. Second, registration or access to the portal does not guarantee use of the portal. A study among 301 patients with asthma reported that patients rarely used the patient portal on a regular basis and only about one-half used the portal in general [23]. In addition, studies cannot possibly take into account all of the interventions/factors other than the patient portal that may contribute to the patient outcomes [23].

Health care systems around the world are adopting patient portals [11,24,25]. In England, the National Health Service (NHS) made patient portals universally available in all general practices since 2015 [26]. The outcomes of the introduction of patient portals in England are yet to be examined, but before any outcomes can be expected, there needs to be an uptake of the technology by patients. Most published systematic reviews about patient portals focused on health outcome [11,24,25], patient experience outcomes such as patient engagement [27] or patient satisfaction [12], facilitators and barriers of use [21], and impact on quality [7]. However, most of these systematic reviews are now outdated due to the increased number of studies about patient portals in the last 1-2 years. Some reviews focused on patient portal adoption by a patient subgroup such as patients with diabetes [6,10], or cancer [2]. One meta-analysis reported patient portal adoption stratified by study setting (controlled versus real-world) but did not report patient portal functionalities of the included studies [21].

Although a number of published systematic reviews about patient portals are available, there are currently no reviews, that we know of, that focus on patient portal functionalities. When describing the outcomes, contextual factors such as the health care setting, patient type, and the functionalities of the patient portal should be considered as much as possible. Research reporting patient portal outcomes can provide valuable evidence if the context and the patient portal functionalities are clearly described and specified [6], because the context could contribute to the success of the technology. In addition, because patient portal adoption can generally be higher among patients with chronic diseases [28], the literature focused on studying the patient portal mostly by patient subpopulations such as those with diabetes or hypertension [17]. It is, therefore, essential to report user characteristics and contextual factors when considering patient portals.

There are a number of systematic review protocols of ongoing reviews about patient portals. Ammenwerth et al. [29] are planning to report patient portal outcomes based on the functionalities of the portal in which they will compare patient portals with access to the EHR alone with portals that have additional functionalities. However, the review will only include randomized controlled trials and cluster randomized controlled trials. Other upcoming reviews are examining patient portal user *expectations* [30]. An upcoming review by Petrovskaya et al. [31] plans to report patient and health care outcomes as a result of using patient portals among others; however, this will be an umbrella review and will only include other systematic reviews. Some other upcoming reviews will only focus on specific diseases such as diabetes, multiple sclerosis, and lower limb arthroplasty [32], or on one functionality of the patient portals (ie, having access to the medical record) [33], or will report only specific health care outcomes such as *no-show appointments* and *emergency visits* [34].

The aim of this systematic review is to synthesize the evidence on health and health care quality outcomes associated with patient portal use among adult (18 years or older) patients.

The review research questions are as follows:

- What kind of health outcomes do tethered patient portals and patient portal functionalities contribute to in adult patients (18 years or older)?
- What kind of health care quality outcomes, including health care utilization outcomes, do tethered patient portals and patient portal functionalities contribute to in adult patients (18 years or older)?

All research questions will be stratified by country, disease type, and health care system model.

## Methods

### Guidelines and Study Registration

This section will outline the methods of the systematic review using the Preferred Reporting Items for Systematic Review and Meta-Analysis Protocols (PRISMA-P) guidelines [35] (Multimedia Appendix 1). This protocol is registered in PROSPERO, International Prospective Register of Systematic Reviews (registration number: CRD42019141131).

### Inclusion Criteria

The review inclusion criteria with reference to participants, interventions, comparators, and outcomes (PICOS) framework [36] are reported in Table 1.

**Table 1:** Inclusion criteria for the systematic review using the participants, interventions, comparators, and outcomes (PICOS) framework.

Characteristics	Criteria
Population	All adult patient(s) (18 years or older).
Intervention	Tethered patient portals only (patient portals that are connected to the patient’s electronic medical record). Patient portals functionalities could include, but are not limited to functionalities for viewing the medical record, making appointments or ordering repeat prescriptions, communicating with the health care providers. Patient portals will be included as long as they are connected to the electronic health care record despite the functionalities they offer. The patient portal could be based in any health care setting including primary care, secondary care, or specialist care.
Comparator	Usual care, other intervention, or no comparator.
Outcomes	Primary outcomes: Changes in patient health outcome measures associated with portal use. Changes in health care quality outcomes including health care utilization.
Study types	Only quantitative study design will be included.

### Exclusion Criteria

- PHRs or patient portals that are not connected to the EHR.
- The review will exclude patient portals that are designed only to deliver patient education or counselling.
- Qualitative studies.

### Information Sources and Search Strategy

The databases that will be searched include (1) MEDLINE (through Ovid), (2) EMBASE (through Ovid), and (3) Scopus. The search will have no restrictions or limits. The base search strategy was developed in the MEDLINE database (Multimedia Appendix 2) through multiple discussions with a medical librarian. The strategy will be modified and adjusted for each database according to the relevant keywords and subject headings in each database. Further studies will be identified through checking the references of eligible studies.

### Study Records and Selection

We will use the Zotero (Roy Rosenzweig Center for History and New Media, USA) reference management software for removing duplicates and managing records. Studies will be scanned through the reading of the titles and abstracts in the first instance. Study title and abstracts will be assessed against the inclusion and exclusion criteria and studies that are clearly irrelevant will be excluded. Relevant studies and studies that did not provide enough detail in the abstract to judge their eligibility will then be assessed through full-text reading. Two members of the research team will independently perform title, abstract, and full-text screenings of the studies and discuss any discrepancies with a third reviewer. The study selection process will be recorded in a PRISMA flow diagram [35].

### Data Extraction

Data extraction will be performed independently by two reviewers. A data extraction form was formulated to collect relevant data from the identified studies (Multimedia Appendix 3). The information collected in the data abstraction form will include (1) the last name of the author(s) and year of publication; (2) country of patient portal; (3) patient portal functionalities (the types of functionalities will not be defined prior to the review to avoid limiting the types that could be included. However, the functionalities, could include, but are not limited to functionalities mentioned in Figure 1, including accessing information through the portal, e-messaging, repeat prescription ordering, or appointment booking); (4) study type (controlled or real world); (5) patient characteristics, such as age group and sex; (6) any other patient characteristics specified in the study (such as patients with diabetes); (7) context-related factors (if any), such as type of health care setting (primary care or secondary care, or private or public health care); and (8) outcomes (health outcomes and health care quality outcomes will be recorded separately). Health outcomes could include any changes in disease indicators such as changes in blood pressure, plasma glucose concentration (hemoglobin A1c), or cholesterol levels. Health care quality outcomes could include any indicators of health care quality such as health care utilization rates, mortality rates, or disease-specific quality indicators. Study type is important because a previous meta-analysis found significant differences in adoption rates between controlled settings and real-world settings with patients being 10.8 times more likely to adopt the portals in controlled settings when compared with real-world settings (95% CI 3.2-36.3) [21].

### Quality Appraisal

The risk of bias of randomized controlled trials will be assessed using the Cochrane Collaboration’s tool for assessing risk of bias in randomized trials [37]. Observational, cross-sectional, and quasi-experimental studies will be assessed using the National Heart, Lung, and Blood Institute (NHLBI) quality assessment tools for each study design [38]. The NHLBI tool helps assess the “internal validity of studies” and identify possible sources of bias [39]. The tool can be used to rate studies as good, fair, or poor in terms of risk of bias. Risk of bias assessment will be performed independently by two reviewers using the criteria from each of the risk of bias assessment tools depending on the type of study (Tables 1 and 2 in Multimedia Appendix 4). Summaries of risk of bias assessments will be presented as a table or a graph or in both formats for each study design. Results of the risk of bias assessment will be taken into account when interpreting findings from the studies; however, these results will not likely be used to exclude studies based on the risk of bias. These results will instead further help assess the confidence in outcomes concluded from the included studies.

### Data Analysis and Synthesis

It is anticipated that performing a meta-analysis will be unlikely due to the heterogeneity of studies. Studies are likely to vary in terms of the methods used to collect and analyze the data, and in the outcomes reported. Alternatively, a narrative synthesis method will be used following guidelines suggested by Popay et al. [40] and the Cochrane Consumers and Communication Review Group (data synthesis and analysis document) [41]. The analysis will start by exploring the differences between and within the studies and identifying patterns in study outcomes. The relationship between studies, and gaps in the study findings will also be addressed in the synthesis.

## Results

This systematic review is ongoing. The software searches started in September 2019. Data abstraction and data synthesis are expected to be completed by the end of June 2020. The review is anticipated to be completed by the end of June 2020. We are planning to disseminate the forthcoming systematic review in a peer-reviewed journal.

## Discussion

### Principal Study Findings

This systematic review will provide a comprehensive overview of the patient portal literature. To make it easy to compare between studies examining patient portals, we will categorize results by patient portal functionalities, disease, country, and health care system model. In the discussion section of the completed review, we will discuss implications from the studies, researcher assumptions, quality of the data, strengths and limitations of the review, and areas for future research.

### Comparison With Previous Work

A common theme in current systematic reviews is the high heterogeneity between studies evaluating or assessing patient portals as well as the high heterogeneity between the functionalities offered by each portal [10]. Certain patient portal functionalities can have a higher association with improved health outcomes than others [6]. To control for the differences between the different portals, we will compare outcomes associated with portals based on the patient portal functionalities taking into account the disease or condition for which the portals are used for whenever possible. The outcomes of this review will inform a population-level analysis of patient portal use stratified by patient portal functionality, disease, country, and health care system model.

### Limitations

It is a possibility that studies with low patient portal uptake do not report or publish their study findings. One way to deal with publication bias is by including both published and unpublished literature such as gray literature [42]. However, due to time constraints this review will only include published and peer-reviewed studies which subjects this review also to publication bias. The review will omit qualitative studies due to the drastic differences in methods and reporting of the results. However, this does not indicate that qualitative studies are not important to understand the outcomes related to patient portals. A systematic review focusing on qualitative studies reporting patient portal outcomes will help to further understand the mechanisms involved between patient portal use and health outcomes and can complement the findings of the forthcoming review.

## Appendix

Multimedia Appendix 1 PRISMA-P 2015 Checklist.

Multimedia Appendix 2 Sample search strategy.

Multimedia Appendix 3 Sample data extraction form.

Multimedia Appendix 4 Sample risk of bias assessment forms.

## Abbreviations

EHR: electronic health record
NHLBI: National Heart, Lung, and Blood Institute
NHS: National Health Service
PHR: personal health record
PICOS: participants, interventions, comparators, and outcomes
PRISMA-P: Preferred Reporting Items for Systematic Review and Meta-Analysis Protocols
