# Supplementary material for: Patient Portal Functionalities and Uptake: Systematic Review Protocol
Source: JMIR Res Protoc. 2020 Jul 31;9(7):e14975. doi: 10.2196/14975 (PMC7428936; doi:10.2196/14975)
Supplement: Multimedia Appendix 2 [file resprot_v9i7e14975_app2.docx]

**Multimedia Appendix 2: Sample search strategy**

Sample search strategy displaying the MEDLINE search through OVID**:**

1. Patient Portals/
2. web portal*
3. electronic personal health record*
4. (patient* adj2 portal*) NOT Vein
5. electronic portal*
6. healthcare portal*
7. health care portal*
8. online portal*
9. personal health record*
10. personal medical record*
11. (Web-based adj1 portal*)
12. personal electronic health record*
13. personal electronic medical record*
14. patient accessible electronic health record*
15. patient accessible electronic medical record*
16. personal health information management system*
17. patient accessible electronic health record*
18. Patient platform*
19. Internet-based patient portal*
20. 1 OR 2 OR 3 OR 4 OR 5 OR 6 OR 7 OR 8 OR 9 OR 10 OR 11 OR 12 OR 13 OR 14 OR 15 OR 16 OR 17 OR 18 OR 19

(No limits)

- *An asterisk helps find the singular and plural variations of the word. For example, portal* will detect both the words portal and portals.

Results: 5770
