# Supplementary material for: Patient Portal Functionalities and Uptake: Systematic Review Protocol
Source: JMIR Res Protoc. 2020 Jul 31;9(7):e14975. doi: 10.2196/14975 (PMC7428936; doi:10.2196/14975)
Supplement: Multimedia Appendix 3 [file resprot_v9i7e14975_app3.docx]

**Multimedia Appendix 3: Sample data extraction form**

| **Author(s) name and publication year** | **Country** | **Patient portal feature(s)** | **Study design (controlled, real-world)** | **Patient Characteristics** | | | | **Healthcare Context** | | **Outcomes** |
| --- | --- | --- | --- | --- | --- | --- | --- | --- | --- | --- |
|  |  |  |  | **Age group** | **sex** | **Patient type (only if specified by the study such as patients with diabetes or patients with hypertension)** | **n** | **Private/Public** | **Other** |  |
| ... | ... | ... |  | ... | ... | ... |  | ... |  | ... |
