# Supplementary material for: Patient Portal Functionalities and Uptake: Systematic Review Protocol
Source: JMIR Res Protoc. 2020 Jul 31;9(7):e14975. doi: 10.2196/14975 (PMC7428936; doi:10.2196/14975)
Supplement: Multimedia Appendix 4 [file resprot_v9i7e14975_app4.docx]

**Multimedia Appendix 4. Sample risk of bias assessment forms**

**Table 1.** Risk of bias assessment form for randomised controlled trials using the Cochrane Collaboration Risk of Bias Tool adapted from: Higgins, J.P.T., et al., *The Cochrane Collaboration’s tool for assessing risk of bias in randomised trials.* BMJ, 2011. **343**: p. d5928.

| **Domain** | **Author's judgement (low, high, unclear)** | **Support for Judgement** |
| --- | --- | --- |
| Selection bias: Random sequence generation |  |  |
| Selection bias: Allocation concealment |  |  |
| Performance bias: Blinding (participants and personnel) |  |  |
| Detection bias: Blinding (outcome assessment) |  |  |
| Attrition bias: Incomplete outcome data |  |  |
| Reporting bias: Selective reporting |  |  |
| Other bias: Other sources of bias |  |  |

**Table 2.** Risk of bias assessment form for observational cohort and cross-sectional studies using the NIH - National Heart, Lung and Blood Institute quality assessment tool adapted from: National Heart, Lung, and Blood Institute (NHLBI). *Study Quality Assessment Tools*. US Department of Health & Human Services 2019 2019-10-15 15:21:09. Available from:<https://www.nhlbi.nih.gov/health-topics/study-quality-assessment-tools>

| **Criteria** | **Answer (Yes, No, Other)** | **Support for Judgement** |
| --- | --- | --- |
| 1. Was the research question or objective in this paper clearly stated? |  |  |
| 2. Was the study population clearly specified and defined? |  |  |
| 3. Was the participation rate of eligible persons at least 50%? |  |  |
| 4. Were all the subjects selected or recruited from the same or similar populations (including the same time period)? Were inclusion and exclusion criteria for being in the study prespecified and applied uniformly to all participants? |  |  |
| 5. Was a sample size justification, power description, or variance and effect estimates provided? |  |  |
| 6. For the analyses in this paper, were the exposure(s) of interest measured prior to the outcome(s) being measured? |  |  |
| 7. Was the timeframe sufficient so that one could reasonably expect to see an association between exposure and outcome if it existed? |  |  |
| 8. For exposures that can vary in amount or level, did the study examine different levels of the exposure as related to the outcome (e.g., categories of exposure, or exposure measured as continuous variable)? |  |  |
| 9. Were the exposure measures (independent variables) clearly defined, valid, reliable, and implemented consistently across all study participants? |  |  |
| 10. Was the exposure(s) assessed more than once over time? |  |  |
| 11. Were the outcome measures (dependent variables) clearly defined, valid, reliable, and implemented consistently across all study participants? |  |  |
| 12. Were the outcome assessors blinded to the exposure status of participants? |  |  |
| 13. Was loss to follow-up after baseline 20% or less? |  |  |
| 14. Were key potential confounding variables measured and adjusted statistically for their impact on the relationship between exposure(s) and outcome(s)? |  |  |
| **Overall rating** |  |  |
